# Accelerometry-Assessed Physical Activity and Circadian Rhythm to Detect Clinical Disability Status in Multiple Sclerosis: Cross-Sectional Study

**DOI:** 10.2196/57599

**Published:** 2025-03-31

**Authors:** Nicole Bou Rjeily, Muraleetharan Sanjayan, Pratim Guha Niyogi, Blake E Dewey, Alexandra Zambriczki Lee, Christy Hulett, Gabriella Dagher, Chen Hu, Rafal D Mazur, Elena M Kenney, Erin Brennan, Anna DuVal, Peter A Calabresi, Vadim Zipunnikov, Kathryn C Fitzgerald, Ellen M Mowry

**Affiliations:** 1 Department of Neurology, Johns Hopkins University School of Medicine, 600 North Wolfe Street, Pathology 627, Baltimore, MD, 21287, United States, 1 4437226779; 2 Department of Biostatistics, Johns Hopkins University Bloomberg School of Public Health, Baltimore, MD, United States

**Keywords:** multiple sclerosis, disability, progressive, physical activity, circadian rhythm, accelerometer, ActiGraph, accelerometry

## Abstract

**Background:**

Tools for measuring clinical disability status in people with multiple sclerosis (MS) are limited. Accelerometry objectively assesses physical activity and circadian rhythmicity profiles in the real-world environment and may potentially distinguish levels of disability in MS.

**Objective:**

This study aims to determine if accelerometry can detect differences in physical activity and circadian rhythms between relapsing-remitting multiple sclerosis (RRMS) and progressive multiple sclerosis (PMS) and to assess the interplay within person between the 2 domains of physical activity (PA) and circadian rhythm (CR) in relation to MS type.

**Methods:**

This study represents an analysis of the baseline data from the prospective HEAL-MS (home-based evaluation of actigraphy to predict longitudinal function in multiple sclerosis) study. Participants were divided into 3 groups based on the Expanded Disability Status Scale (EDSS) criteria for sustained disability progression: RRMS-Stable, RRMS-Suspected progression, and PMS. Baseline visits occurred between January 2021 and March 2023. Clinical outcome measures were collected by masked examiners. Participants wore the GT9X Link ActiGraph on their nondominant wrists for 2 weeks. After adjusting for age, sex, and BMI, a logistic regression model was fitted to evaluate the association of each accelerometry metric with odds of PMS versus RRMS. We also evaluated the association of accelerometry metrics in differentiating the 2 RRMS subtypes. The Joint and Individual Variation Explained (JIVE) model was used to assess the codependencies between the PA and CR domains and their joint and individual association with MS subtype.

**Results:**

A total of 253 participants were included: 86 with RRMS-Stable, 82 with RRMS-Suspected progression, and 85 with PMS. Compared to RRMS, participants with PMS had lower total activity counts (β=−0.32, 95% CI −0.61 to −0.03), lower time spent in moderate to vigorous physical activity (β=−0.01, 95% CI −0.02 to −0.004), higher active-to-sedentary transition probability (β=5.68, 95% CI 1.86-9.5), lower amplitude (β=−0.0004, 95% CI −0.0008 to −0.0001), higher intradaily variability (β=4.64, 95% CI 1.45-7.84), and lower interdaily stability (β=−4.43, 95% CI −8.77 to −0.10). Using the JIVE model for PA and CR domains, PMS had higher first joint component (β=0.367, 95% CI 0.088-0.656), lower PA-1 component (β=−0.441, 95% CI −0.740 to −0.159), and lower PA-2 component (β=−0.415, 95% CI −0.717 to −0.126) compared to RRMS. No significant differences were detected between the 2 RRMS subtypes except for lower relative amplitude in those with suspected progression (β=−5.26, 95% CI −10.80 to −0.20).

**Conclusions:**

Accelerometry detected differences in physical activity patterns between RRMS and PMS. More advanced analytic techniques may help discern differences between the 2 RRMS subgroups. Longitudinal follow-up is underway to assess the potential for accelerometry to detect or predict disability progression.

## Introduction

Multiple sclerosis (MS) is a chronic disease affecting the central nervous system in which those affected typically have intermittent neurologic symptoms and signs early in the course (relapsing-remitting multiple sclerosis [RRMS]) but often, subsequently (or, less commonly, from onset), slowly accumulate disability (progressive multiple sclerosis [PMS]). Currently available tools to measure disability in MS are limited. The Expanded Disability Status Scale (EDSS) is the most widely used clinical outcome measure and is considered by the Food and Drug Administration to be the gold standard for phase 3 trials in PMS. The EDSS, however, is semiquantitative, has limited reliability, and only captures a person’s state at one short point in time during a clinical visit [1]. People with MS may have different disability states at different times throughout the day, as symptoms can fluctuate with temperature, fatigue, stress, and other factors [2,3]. The limitations of the EDSS lead to long delays in confirming that a person has transitioned from RRMS to PMS and also inflate the sample size and follow-up time required for MS trials for which disability progression is the primary end point [4,5].

Triaxial accelerometry is a safe and relatively inexpensive tool that may offer an objective and sensitive measure of disability in people with MS. With the use of an accelerometer worn on the wrist, real-time information about physical activity and circadian rhythmicity patterns can be collected in a person’s natural environment. Such data may allow detection of variation in activity that may be missed during clinical visits [6,7]. Several studies have looked at the use of accelerometry in MS to identify associations with sleep, fatigue, depression, or disability outcomes (EDSS and Patient-Determined Disease Steps); however, whether wrist-worn accelerometry can differentiate between people with RRMS and PMS remains an existing question [8-16]. We hypothesized that people with RRMS and PMS have different physical activity and circadian rhythmicity patterns. Due to the interplay between physical activity and circadian rhythm domains within person, we also aimed to study joint and individual variations in these 2 domains and their associations with MS subtype.

## Methods

### Participant Selection and Study Design

This study includes an analysis of the baseline data from the longitudinal observational study HEAL-MS (home-based evaluation of actigraphy to predict longitudinal function in multiple sclerosis; Figure S1 in Multimedia Appendix 1). Eligible participants followed at the Johns Hopkins Multiple Sclerosis Precision Medicine Center of Excellence were recruited between January 2021 and March 2023. Participants had to have a diagnosis of MS, were aged ≥40 years, had no apparent comorbidities that may limit physical activity (eg, heart failure and end-stage renal disease), had no MS relapse within the last 6 months prior to enrollment, and had baseline EDSS score ≤6.5. These criteria were chosen so that the participants with RRMS were of the age at which transition to PMS might begin within the next few years, the changes in accelerometry measures were more likely to be due to MS and not comorbid conditions, and the baseline measures were not affected by recovery from a recent relapse. Medical records were reviewed by an MS-trained neurologist to ensure eligibility.

Participants were divided into 3 groups with a target of 85 participants each: those with stable RRMS who had no suspected or confirmed progression (RRMS-Stable), those with PMS who had confirmed disability worsening on EDSS (PMS), and those with RRMS who were suspected to be progressing clinically (inferred by an MS-trained neurologist from medical record notes or personal knowledge of the participant) but did not have sustained disability worsening on exam (RRMS-Suspected progression). Confirmed disability worsening on exam was defined as an EDSS change of ≥1.0 point if baseline EDSS was ≤5.5, or of ≥0.5 points if baseline EDSS was ≥6.0; this change should be sustained for ≥24 weeks, not in the context of an explanatory relapse. The 2 RRMS groups were matched based on age (target ±2 years with prespecified ability to relax the criterion if necessary), sex at birth, race or ethnicity, and efficacy class of current disease-modifying therapy (no treatment, first-line treatment [injectables and oral therapies (except for cladribine or ofatumumab)], or higher-efficacy treatment [infusion therapies and cladribine]). The PMS group was expected to be a little older and to be prescribed different (or even no) medications due to the likely lack of neuroinflammation and relapse activity in this group. However, enrollment of a comprehensive cohort of participants with respect to sex and race or ethnicity was attempted.

### Clinical Measures

The EDSS exam was conducted after the fitting of the accelerometer and was performed by a masked EDSS-trained physician. A modified multiple sclerosis functional composite (MSFC), which includes the 9-hole peg test, the timed 25-foot walk test, the high- and low-contrast letter acuity (binocular, 2.5% contrast Sloan charts), and the Symbol Digit Modalities Test, was performed by a masked, trained study team member. Participants also completed patient-reported outcomes such as the International Physical Activity Questionnaire (IPAQ).

### Accelerometry Measures

Accelerometry metrics were derived from accelerometry data collected with the GT9X Link ActiGraph using a built-in triaxial accelerometer [17]. All participants were instructed to wear the ActiGraph device on the wrist of their nondominant hand for 24 hours a day over a duration of 2 weeks. Accelerometers were set to capture 3D acceleration at 30 Hz with the acceleration range of ±8 G. The raw acceleration data (.gt3x) were downloaded from the device using ActiLife v6.134 Lite Edition. Binary raw activity data (Hz-level accelerometry data) were read by read.gt3x package into an R data frame (R Foundation for Statistical Computing) and transformed into 60-second epochs activity count data in 1440 minutes per day (12 AM to 11:59 PM) analytic format. The activity counts are vector magnitude-based activity counts.

The following criteria were applied to define valid days: (1) intervals of 90 minutes or longer with all minute-level activity counts equal to 0 were defined as nonwear intervals [18], (2) valid days were defined as those with total wear-time ≥90% of the day (≥1296 minutes of wear), and (3) each participant should have ≥3 valid days of accelerometry data.

When a wear period overlapped with the transition to or from daylight saving time, the following adjustments were made. If the clock was set 1 hour forward, 1 hour of data were missing, so imputation was used by averaging the same hour from other valid days for this participant. If the clock was set 1 hour back, duplicate data were generated for that hour; in this instance, we used the average of the duplicated hour, and the loss of 1 hour on the final day was imputed by averaging the same hour from the other valid days for this participant. The wear period overlapped with the transition to or from daylight saving time for only 5 participants.

Accelerometry measures in this study are presented as averages over all valid days and included (1) measures of volume and intensity: total activity count (TAC; daily and 2-hour specific), total log-transformed activity count (TLAC), the total daily number of nonactive minutes, the number of minutes spent in moderate to vigorous physical activity (MVPA), and the number of minutes spent in light intensity physical activity (LIPA); (2) measures of composition: MVPA/LIPA (the ratio of the time spent in MVPA over the time spent in LIPA) and MVPA/nonactivity (the ratio of the time spent in MVPA over the time spent nonactive); (3) measures of fragmentation: sedentary-to-active transition probability (SATP) and active-to-sedentary transition probability (ASTP) [19,20]; and (4) diurnal landmarks of rest-activity rhythms: average log acceleration during the most active 10 hours of the day (M10), midpoint of M10, average log acceleration during the least active 5 hours of the day (L5), midpoint of L5, daytime activity ratio estimate (DARE), amplitude, relative amplitude (RA), midline estimating statistic of rhythm (MESOR), acrophase, intradaily variability (IV), and interdaily stability (IS) [21].

The following sample-specific cut points were developed for discriminating between nonactive, LIPA, and MVPA minutes: (1) nonactive minute was defined as a minute with activity counts ≤2000, (2) LIPA minute was defined as activity counts >2000 and ≤6750, and (3) MVPA minute was defined as activity counts >6750. These cut points were specific to our cohort and defined based on an internal calibration procedure that maximized concordance between accelerometry-based estimates and the participants’ self-reported physical activity outcomes in the IPAQ (Table S1 and Figure S2 in Multimedia Appendix 1 [22]). Our derived cut points were similar to those defined in other population cohorts [22-24]. Rather than using predetermined cutoffs reported in the literature, we believe this method was more suitable for this study due to different cohort demographics and clinical conditions that may alter average physical activity intensity.

### Statistical Analyses

All statistical analyses were conducted using R (version 4.3.1; R Foundation for Statistical Computing). Descriptive statistics of the demographics, clinical data, and accelerometry measures for each of the 3 groups were summarized as mean (SD) or median (IQR). Differences between the combined RRMS group (RRMS-Stable and RRMS-Suspected progression) and PMS group were compared using *t* tests and Pearson *χ*² test as appropriate. After adjusting for age, sex, and BMI, a logistic regression model was fitted to evaluate the association of each accelerometry metric with odds of PMS (vs RRMS). We also evaluated the association of accelerometry metrics in differentiating the RRMS subtypes (RRMS-Suspected progression vs RRMS-Stable). To account for differences in duration and timing of sleep, sensitivity analyses were performed by re-exploring the same accelerometry-derived measures within the most active 10 hours of each day (M10) rather than the 24-hour daily total.

### Functional Principal Component Analysis

Functional principal component (fPC) analysis, applied to diurnal rest-activity rhythms (minute-level activity profiles), captures main patterns of temporal allocation of activity and provides important diurnal landmarks. The choice of the first 5 fPCs as measures of circadian rhythmicity is based on 2 factors. First, they capture most of the diurnal variability (76%) in our data. Second, the use of fPC analysis in accelerometry studies of diurnal rest-activity rhythms in large national cohorts such as the National Health and Nutrition Examination Survey (NHANES) and the UK Biobank mostly focuses on the first 4 fPCs since functional principal components beyond the 4th typically capture a relatively low proportion of variance in the data, and their shapes tend to be very difficult to interpret due to their very dynamic shape [25-27]. Nevertheless, in our case, the fifth fPC explained a significant proportion of the total variability (4%) and was also included. Figure S3 in Multimedia Appendix 1 shows that the additional fPCs beyond the 5th did not provide meaningful explanations of variability.

### Joint and Individual Variation Explained

**Figure 1.**
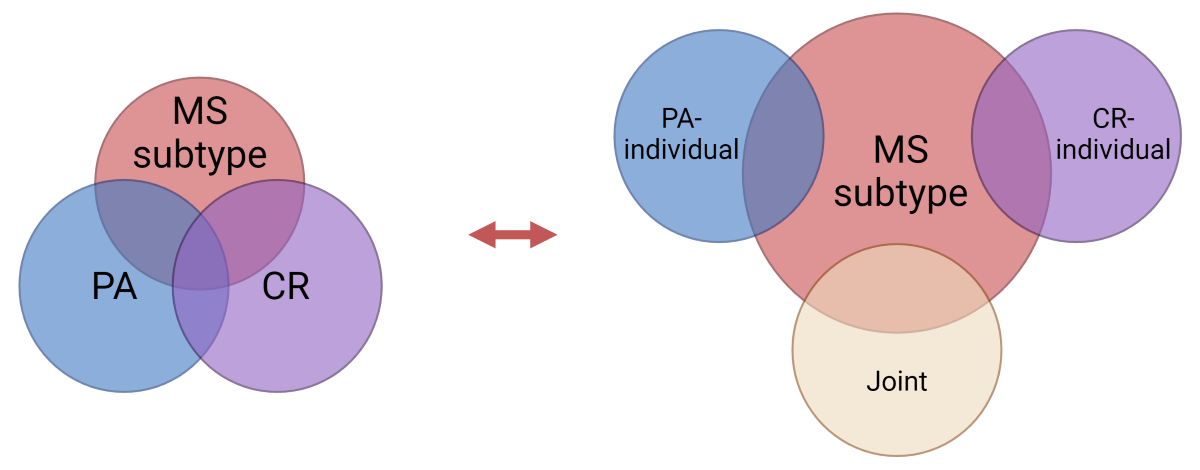
Conceptual representation of associations between multiple sclerosis (MS) subtype and the physical activity (PA) and circadian rhythm (CR) domains performed in standard regression modeling (left) versus the associations between MS subtype and 3 sets of independent (uncorrelated) latent variables representing joint-PA-CR, individual-PA, and individual-CR information after JIVE (Joint and Individual Variation Explained) decomposition (right). Regression analysis with JIVE components as predictors can reveal and distinguish joint and individual associations between the PA and CR domains and MS subtype.

The Joint and Individual Variation Explained (JIVE) is an integrative dimension reduction technique that can be applied to multiple features grouped within several domains [28]. The physical activity (PA) domain was characterized by measures of total volume of physical activity, times spent in different intensities of activity and composition ratios of those times, measures of activity fragmentation/continuity, and temporal-local measures of total activity presented in 2-hour bins. The circadian rhythmicity (CR) domain was characterized by measures of the strength of circadian rhythms (RA and related components such as M10 and timing of M10), measures of variability including IS and IV, parametric measures extracted from the cosinor model (MESOR, amplitude, acrophase), and the first 5 fPCs. There is a significant amount of interdependence between the PA and CR domains since the measures belong to the same participants over the same time frame. Variations in PA and CR domains that are independent from each other are also expected. JIVE is a model developed to explore such situations and to decompose the joint and individual effects [29]. Figure 1 demonstrates the conceptual representation of the benefits of JIVE to better understand the association between MS subtype and the joint and individual variation of PA and CR domains. We applied JIVE to the domains of PA and CR to create three groups of latent variables that explain (1) joint variation shared across PA and CR, (2) individual variation specific to each of the domains, and (3) remaining unexplained variation.

### Ethical Considerations

The study was approved by the institutional review board at Johns Hopkins University (IRB00243681). All participants provided written informed consent and had the ability to opt out at any point. All data are stored on REDCap (Research Electronic Data Capture), and only study members who are approved by the institutional review board have access to the data. The data are deidentified to the greatest extent possible. All participants received US $40 as compensation for the baseline visit to offset study-related travel and parking costs. All study procedure costs, including shipping of the ActiGraph, were covered as part of the study.

## Results

### Demographics and Clinical Outcomes

A total of 275 people with MS completed a baseline study visit as part of the HEAL-MS study; 253 had retrievable accelerometry data and were included in this baseline analysis (16 never returned the device, while 6 had insufficient valid days). Participants were divided into three groups: 86 with RRMS-Stable, 82 with RRMS-Suspected progression, and 85 with PMS. Baseline visits occurred between January 2021 and March 2023. Table 1 shows the demographic and clinical data for the 3 groups.

**Table 1.** Demographics and clinical data collected at the baseline visit for HEAL-MS^a^ participants.

		RRMS^b^		PMS^c^ (n=85)	*P* value^d^
		RRMS-Stable (n=86)	RRMS-Suspected progression (n=82)		
Demographics					
Age (years), mean (SD)		53 (7)	54 (8)	57 (9)	<.001^e^
Sex (female), n (%)		61 (71)	61 (74)	57 (67)	.36
Race, n (%)					.67
White		64 (74)	63 (77)	67 (79)	
Black		19 (22)	14 (17)	13 (15)	
Other		3 (4)	5 (6)	5 (6)	
Hispanic or Latino, n (%)		4 (5)	3 (4)	3 (4)	.79
BMI, mean (SD)		29.4 (6.4)	28.2 (5.6)	28 (6.2)	.35
Employment status, n (%)					.18
Full time		45 (58)	37 (51)	32 (43)	
Part time		4 (5)	6 (8)	7 (9)	
Homemaker		7 (9)	2 (3)	4 (5)	
Retired		13 (17)	13 (18)	18 (24)	
Unemployed		3 (4)	3 (4)	0 (0)	
Disability		6 (8)	12 (16)	14 (19)	
Clinical data					
EDSS^f^ score, median (IQR)		2.0 (1.5-3.0)	3.0 (2.0-3.5)	5.0 (3.5-6.0)	<.001^e^
MSFC^g^, median (IQR)					
25-foot walk		4.5 (4.0-5.1)	5.3 (4.3-6.2)	7.2 (5.8-9.5)	<.001^e^
D^h^-9hpg^i^		21.3 (19.4-24.0)	23.8 (20.9-27.3)	26.9 (22.1-34.4)	<.001^e^
ND^j^-9hpg		22.7 (20.8-25.2)	25.5 (21.9-28.9)	27.4 (23.5-34.6)	<.001^e^
SDMT^k^		51 (47-59)	48 (38-57)	42 (34-50)	<.001^e^
Tremor^l^, median (IQR)		0 (0-0.5)	0 (0-0.5)	0 (0-1)	.20

a HEAL-MS: home-based evaluation of actigraphy to predict longitudinal function in multiple sclerosis.

b RRMS: relapsing-remitting multiple sclerosis.

c PMS: progressive multiple sclerosis.

d *P* value from *t* test or Pearson *χ*² test comparing RRMS (RRMS-Stable and RRMS-Suspected progression) vs PMS.

e *P*<.05

f EDSS: Expanded Disability Status Scale.

g MSFC: multiple sclerosis functional composite.

h D: dominant hand.

i 9hpg: 9-hole peg test.

j ND: nondominant hand.

k SDMT: Symbol Digit Modalities Test.

l Bain Score for Tremor Severity (BSTS) was used for tremor evaluation.

### Accelerometry Measures Over 24 Hours

**Figure 2.**
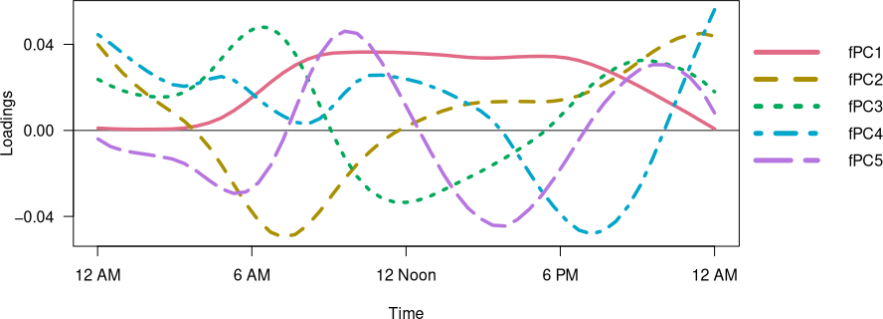
First 5 functional principal components (fPCs). fPC 2, 3, and 4 values are multiplied by −1.

**Figure 3.**
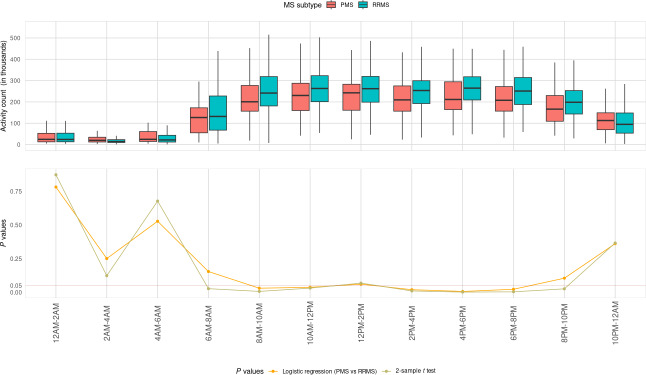
Total activity counts (TAC) for each 2-hour interval over the course of 24 hours for PMS versus RRMS. The 2-sample *t* test *P* values show the 2-hour intervals when TAC was significantly different between the 2 groups. As an additional reference, the logistic regression *P* values are shown with the TAC 2-hour intervals used as predictors, after adjusting for age, sex, and BMI. MS: multiple sclerosis; PMS: progressive multiple sclerosis; RRMS: relapsing-remitting multiple sclerosis.

Figure S4 in Multimedia Appendix 1 shows the raw accelerometry data for 1 participant over the course of the week. The fPCs captured important temporal or diurnal landmarks (Figure 2) and are comparable to those captured in large national surveys such as UK Biobank and NHANES [25,27]. Accelerometry metrics for the 3 groups are shown in Table 2. TAC and MVPA, the 2 main measures of daily physical activity volume, as well as M10, amplitude, and MESOR, measures of circadian rhythmicity, were significantly different between the RRMS and PMS groups, with greater intensity of activity in the RRMS groups compared to the PMS group. ASTP was also significantly different, with higher values (more frequent transitions to sedentary time) in the PMS group. When TAC was divided into 2-hour windows, it was significantly lower in the PMS group at specific times of the day (6 AM to 12 PM and 2 PM to 10 PM; Figure 3). Figure S5 in Multimedia Appendix 1 demonstrates the correlation plot for all accelerometry measures; the green box demonstrates the temporal profiles of the circadian measures associated with TAC over 24 hours. MESOR, amplitude, and fPC1 are highly correlated with TAC between 9 AM and 8 PM.

**Table 2.** Mean (SD) baseline accelerometry metrics over 24 hours for HEAL-MS^a^ participants.

	RRMS^b^		PMS^c^ (n=85)	*P* value^d^
	RRMS-Stable (n=86)	RRMS-Suspected progression (n=82)		
Metrics of physical activity volume and fragmentation				
TAC^f^ (x10^3^)	2113 (566)	2181 (724)	1909 (717)	.008^e^
TLAC^g^	6277 (868)	6278 (933)	6196 (999)	.51
Nonactive minutes	1067 (102)	1052 (114)	1087 (130)	.08
MVPA^h^	59 (34)	61 (52)	41 (43)	*<*.001^e^
LIPA^i^	314 (84)	327 (83)	312 (105)	.52
SATP^j^	0.09 (0.03)	0.09 (0.03)	0.09 (0.03)	.21
ASTP^k^	0.27 (0.06)	0.26 (0.06)	0.30 (0.11)	.003^e^
Metrics of circadian rhythm				
M10^l^	2482 (665)	2540 (844)	2161 (806)	*<.*001^e^
Midpoint of M10^m^	2:20 PM (85)	2:15 PM (86)	2:14 PM (77)	.72
L5^n^	111 (67)	135 (109)	146 (124)	.09
Midpoint of L5^m^	3:58 AM (68)	3:58 AM (80)	4:05 AM (76)	.57
RA^o^	0.91 (0.05)	0.89 (0.07)	0.87 (0.09)	*<.*001^e^
DARE^p^	0.70 (0.05)	0.70 (0.05)	0.69 (0.05)	.049^e^
Amplitude	2290 (1081)	2218 (847)	1923 (839)	.008^e^
MESOR^q^	1313 (623)	1264 (390)	1120 (429)	.01^e^
Acrophase	14.56 (1.92)	14.61 (1.35)	14.76 (1.31)	.40
IV^r^	0.52 (0.11)	0.49 (0.09)	0.49 (0.08)	.02^e^
IS^s^	0.26 (0.07)	0.27 (0.07)	0.25 (0.06)	.08
fPC1^t^	1912 (17,574)	4242 (22,747)	−5206 (21,441)	.003^e^
fPC2	933 (11,249)	−1596 (11,922)	507 (10,181)	.59
fPC3	235 (9505)	−166 (8702)	−108 (6432)	.89
fPC4	−1489 (6339)	−585 (8694)	451 (4989)	.10
fPC5	49 (6677)	−489 (5772)	677 (5744)	.27

a HEAL-MS: home-based evaluation of actigraphy to predict longitudinal function in multiple sclerosis.

b RRMS: relapsing-remitting multiple sclerosis.

c PMS: progressive multiple sclerosis.

d *P* value from *t* test comparing RRMS (RRMS-Stable and RRMS-Suspected progression) vs PMS.

e *P*<.05

f TAC: total activity count.

g TLAC: total log-transformed activity count.

h MVPA: moderate to vigorous physical activity.

i LIPA: light intensity physical activity.

j SATP: sedentary-to-active transition probability.

k ASTP: active-to-sedentary transition probability.

l M10: average log acceleration during the most active 10 hours of the day.

m Midpoints of M10 and L5 are represented as time of the day in hour:minutes (±SD in minutes).

n L5: average log acceleration during the least active 5 hours of the day.

o RA: relative amplitude.

p DARE: daytime activity ratio estimate.

q MESOR: midline estimating statistic of rhythm.

r IV: intradaily variability.

s IS: interdaily stability.

t fPC: functional principal component.

In multivariable-adjusted models (Table 3), participants with PMS had lower TAC (β=−0.32, 95% CI −0.61 to −0.03; *P*=.03), lower MVPA (β=−.01, 95% CI −0.02 to −0.004; *P*=.004), lower M10 (β=−0.0006, 95% CI −0.001 to −0.0002; *P*=.003), and higher ASTP (β=5.68, 95% CI 1.86-9.50; *P*=.004) when compared to those with RRMS. PMS (vs combined RRMS) was also associated with lower TAC from 8 AM to 12 PM and from 2 PM to 8 PM (Figure 3). Lower values of compositional ratios MVPA/LIPA and MVPA/nonactive were also associated with PMS (vs RRMS), indicating that individuals with PMS tend to spend relatively less time in moderate/vigorous activity compared to the time in light intensity activity or nonactivity (Table 3). Participants with PMS also had lower amplitude (β=−0.0004, 95% CI −0.0008 to −0.0001; *P*=.01), lower MESOR (β=−0.0009, 95% CI −0.002 to −0.0002; *P*=.01), higher IV (β=4.64, 95% CI 1.45-7.84; *P*=.004), lower IS (β=−4.43, 95% CI −8.77 to −0.10; *P*=.045), and lower fPC1 (β=−0.39, 95% CI −0.69 to −0.09; *P*=.01) (Table 3). There were no significant associations of accelerometry measures between RRMS subgroups except for a difference in RA (β=−5.26, 95% CI −10.80 to −0.20; *P*=.049), with lower RA in RRMS-Suspected progression (Table 3; Figure S6 in Multimedia Appendix 1).

**Table 3.** Multivariable logistic regression model to evaluate the association of each accelerometry metric with multiple sclerosis subtype. The multivariable logistic regression models were adjusted for age, sex, and BMI.

	PMS^a^ vs RRMS^b^ (reference group)			RRMS-Suspected progression vs RRMS-Stable (reference group)		
	Estimate	*P* value	95% CI	Estimate	*P* value	95% CI
TAC^d,e^	−0.32	.03^c^	−0.61 to −0.03	0.14	.42	−0.20 to 0.50
TLAC^d,f^	−0.04	.79	−0.31 to 0.23	0.05	.77	−0.28 to 0.38
Nonactive minutes	0.002	.16	−0.0007 to 0.004	−0.002	.29	−0.005 to 0.001
MVPA^g^	−0.01	.004^c^	−0.02 to −0.004	0.002	.70	−0.006 to 0.009
LIPA^h^	−0.0006	.69	−0.004 to 0.002	0.002	.25	−0.002 to 0.006
SATP^i^	−3.004	.59	−13.84 to 7.83	1.60	.81	−11.16 to 14.47
ASTP^j^	5.68	.004^c^	1.86 to 9.50	−4.44	.13	−10.47 to 1.05
MVPA/LIPA	−4.25	.002^c^	−6.99 to −1.51	−0.31	.81	−2.91 to 2.27
MVPA/nonactive	−7.90	.02^c^	−14.53 to −1.27	2.35	.46	−3.73 to 9.04
LIPA/nonactive	−0.36	.74	−2.50 to 1.78	1.74	.22	−0.98 to 4.58
M10^k^	−0.0006	.003^c^	−0.001 to −0.0002	0.0001	.63	−0.0003 to 0.0005
Midpoint of M10	−0.0004	.82	−0.004 to 0.003	−0.0006	.75	−0.004 to 0.003
L5^l^	0.002	.11	−0.0005 to 0.005	0.004	.07	0 to 0.008
Midpoint of L5	0.0009	.71	−0.004 to 0.005	−0.004	.16	−0.01 to 0.002
RA^m^	−6.00	.002^c^	−9.73 to −2.26	−5.26	.049^c^	−10.80 to −0.20
DARE^n^	−5.80	.04^c^	−11.25 to −0.34	−0.34	.91	−6.42 to 5.74
Amplitude	−0.0004	.01^c^	−0.0008 to −0.0001	−0.0001	.59	−0.0004 to −0.0002
MESOR^o^	−0.0009	.01^c^	−0.002 to −0.0002	−0.0002	.48	−0.0009 to 0.0004
Acrophase	0.08	.37	−0.10 to 0.27	0.02	.84	−0.17 to 0.20
IV^p^	4.64	.004^c^	1.45 to 7.84	−0.30	.88	−4.21 to 3.62
IS^q^	−4.43	.045^c^	−8.77 to −0.10	0.53	.82	−3.97 to 5.03
fPC1^r^	−0.39	.01^c^	−0.69 to −0.09	0.14	.39	−0.19 to 0.47
fPC2	0.04	.79	−0.23 to 0.31	−0.25	.12	−0.58 to 0.07
fPC3	0.08	.57	−0.19 to 0.35	0.009	.96	−0.30 to 0.32
fPC4	0.19	.18	−0.08 to 0.46	0.11	.51	−0.21 to 0.42
fPC5	0.15	.27	−0.12 to 0.42	−0.05	.74	−0.36 to 0.26

a PMS: progressive multiple sclerosis.

b RRMS: relapsing-remitting multiple sclerosis.

c *P*<.05.

d Regression inputs were scaled by dividing TAC and TLAC values by 1 SD.

e TAC: total activity count.

f TLAC: total log-transformed activity count.

g MVPA: moderate to vigorous physical activity.

h LIPA: light intensity physical activity.

i SATP: sedentary-to-active transition probability.

j ASTP: active-to-sedentary transition probability.

k M10: average log acceleration during the most active 10 hours of the day.

l L5: average log acceleration during the least active 5 hours of the day.

m RA: relative amplitude.

n DARE: daytime activity ratio estimate.

o MESOR: midline estimating statistic of rhythm.

p IV: intradaily variability.

q IS: interdaily stability.

r fPC: functional principal component.

### Accelerometry Measures Within M10

To account for differences in duration and timing of sleep, the accelerometry metrics were explored within the M10 period. The results are summarized in Table S2 in Multimedia Appendix 1. TAC, MVPA, and ASTP remained significantly different between the RRMS and PMS groups. Additionally, the PMS group displayed more nonactive time compared to the RRMS group. Logistic regression identified significant associations between the MS subtype (PMS vs RRMS) and TAC, MVPA, nonactive minutes, ASTP, and MVPA/LIPA (Table S3 in Multimedia Appendix 1). No significant associations were identified when comparing the 2 RRMS subgroups.

### The JIVE Components

**Figure 4.**
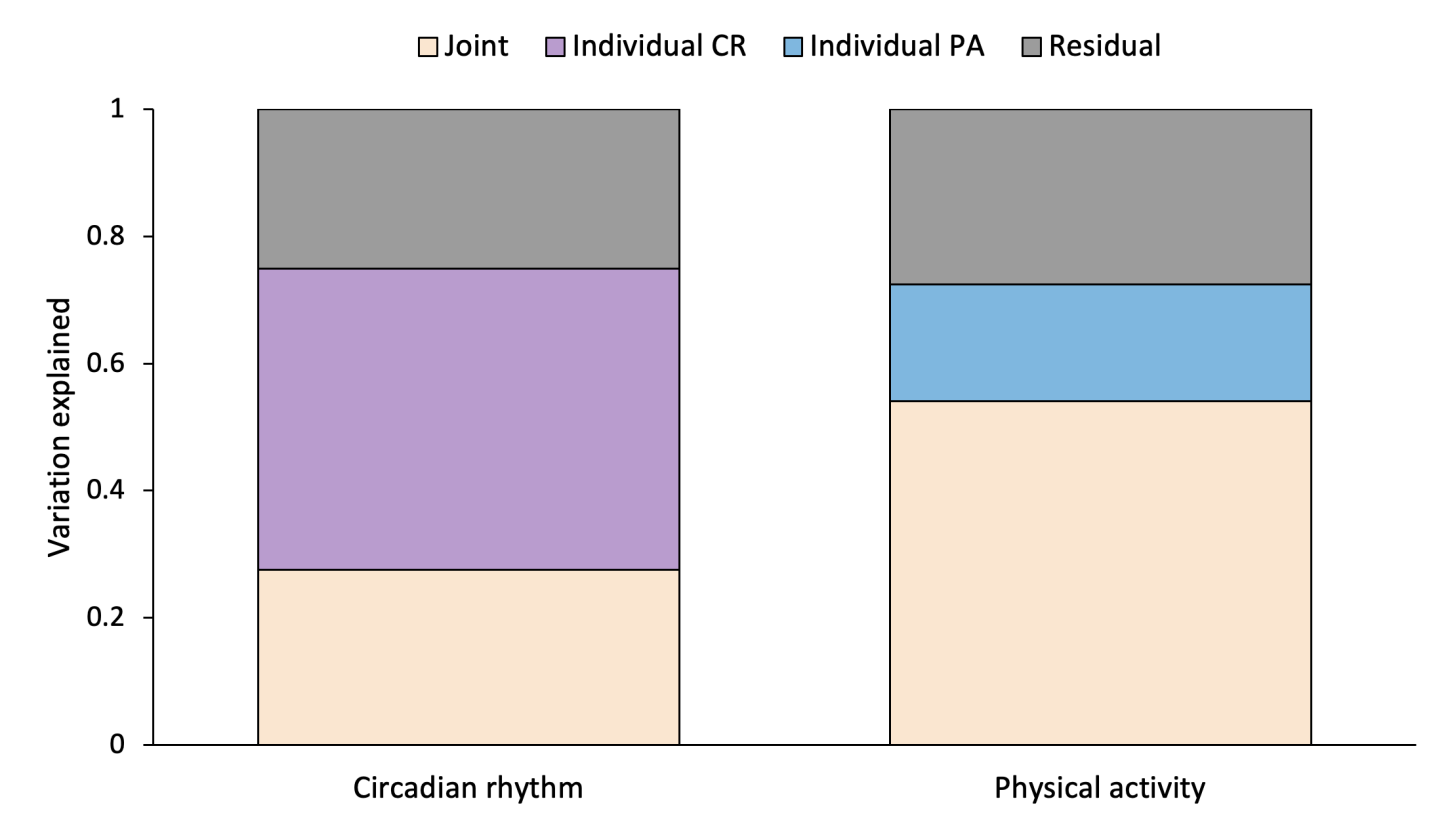
Joint and Individual Variation Explained (JIVE) by the 2 JIVE accelerometry-derived domains of circadian rhythm (CR) and physical activity (PA).

We also applied JIVE, a novel integrative dimension reduction technique. Figure 1 presents the conceptualized diagram of JIVE decomposition and how JIVE can be used to better understand codependencies between PA and CR domains and their joint and individual association with MS subtype. Figure 4 shows the estimated JIVE components obtained from the accelerometry-derived domains of PA and CR. The directions of loadings (+ or −) and squares of loadings for the estimated JIVE joint component and individual PA and CR components are shown in Tables4  5, and Table S4 in Multimedia Appendix 1, respectively. Higher positive scores of the first joint JIVE component primarily represent lower volume of physical activity (captured by TAC) and weaker circadian rhythms (captured by M10, MESOR, amplitude, and fPC1) (Table 4). Higher positive individual PA-1 scores capture less frequent transitions to active behavior (SATP), less time spent in LIPA (LIPA, LIPA/nonactive, TLAC), and more time spent nonactive. Higher positive individual PA-2 scores capture more time spent in MVPA (MVPA, MVPA/LIPA, MVPA/nonactive) and more activity in the morning and evening hours (TAC 8 to 10 AM and TAC 6 PM to 12 AM) (Table 5). Joint JIVE component and individual PA-1 and PA-2 components were found to be significantly associated with MS subtype (Table 6). No significant associations were identified when comparing the 2 RRMS subgroups.

**Table 4.** JIVE^a^ joint component with variables with more than 5% of proportional variation.

Variable	Joint component loading	Relative magnitude of joint component loading
M10^b^	−0.2665385	0.0710428
fPC1^c^	−0.2608276	0.0680310
MESOR^d^	−0.2519508	0.0634792
Amplitude	−0.2408992	0.0580324
TAC^e^	−0.2256510	0.0509184

a JIVE: Joint and Individual Variation Explained.

b M10: average log acceleration during the most active 10 hours of the day.

c fPC: functional principal component.

d MESOR: midline estimating statistic of rhythm.

e TAC: total activity count.

**Table 5.** Individual JIVE^a^ components of physical activity with variables with more than 5% of proportional variation.

Variable	PA component loading	Relative magnitude of PA component loading
Physical activity component 1		
SATP^b^	−0.4493703	0.2019337
LIPA^c^	−0.4141149	0.1714912
LIPA/nonactive	−0.3664975	0.1343204
TLAC^d^	−0.3365927	0.1132946
MVPA/LIPA^e^	0.3293182	0.1084505
Nonactive minutes	0.2448185	0.0599361
Physical activity component 2		
MVPA/LIPA	0.4728996	0.2236340
MVPA	0.3970161	0.1576218
MVPA/nonactive	0.3673462	0.1349432
TAC^f^ 8 PM to 10 PM	0.3341579	0.1116615
TAC 8 AM to 10 AM	0.3099402	0.0960629
TAC 10 PM to 12 AM	0.2477134	0.0613620
TAC 6 PM to 8 PM	0.2430202	0.0590588
Physical activity component 3		
TAC 12 AM to 2 AM	0.6035690	0.3642955
TAC 10 PM to 12 AM	0.4550918	0.2071086
TAC 4 AM to 6 AM	−0.3500618	0.1225433
TAC 2 AM to 4 AM	−0.3085089	0.0951777
Physical activity component 4		
ASTP^g^	−0.5134849	0.2636667
TAC 8 PM to 10 PM	0.3803549	0.1446698
TAC 2 PM to 4 PM	−0.3588593	0.1287800
SATP	−0.3283307	0.1078011
TLAC	−0.2739182	0.0750312
TAC 10 AM to 12 PM	0.2516689	0.0633372

a JIVE: Joint and Individual Variation Explained.

b SATP: sedentary-to-active transition probability.

c LIPA: light intensity physical activity.

d TLAC: total log-transformed activity count.

e MVPA: moderate to vigorous physical activity.

f TAC: total activity count.

g ASTP: active-to-sedentary transition probability.

**Table 6.** Multivariable logistic regression model to evaluate the association of each JIVE^a^ component with multiple sclerosis subtype. The multivariable logistic regression models were adjusted for age, sex, and BMI.

	PMS^b^ vs RRMS^c^ (reference group)			RRMS-Suspected progression vs RRMS-Stable (reference group)		
	Estimate	*P* value	95% CI	Estimate	*P* value	95% CI
Joint component	0.3665	.01^d^	0.0881 to 0.6558	−0.1266	.44	−0.4525 to 0.1897
PA-1^e^	−0.441	.003^d^	−0.7397 to −0.159	−0.0183	.91	−0.3404 to 0.3034
PA-2	−0.4147	.006^d^	−0.7174 to −0.1255	−0.3066	.07	−0.6502 to 0.0207
PA-3	−0.0527	.71	−0.3282 to 0.2204	−0.2622	.11	−0.5887 to 0.0529
PA-4	0.235	.11	−0.046 to 0.5272	0.1849	.25	−0.129 to 0.5098
CR-1^f^	0.2588	.07	−0.0162 to 0.5411	0.0107	.95	−0.3044 to 0.3275
CR-2	−0.2732	.052	−0.5536 to 0.0001	−0.2991	.07	−0.6344 to 0.019
CR-3	−0.142	.32	−0.4266 to 0.135	0.0815	.61	−0.2345 to 0.4031

a JIVE: Joint and Individual Variation Explained.

b PMS: progressive multiple sclerosis.

c RRMS: relapsing-remitting multiple sclerosis.

d *P*<.05.

e PA: individual physical activity component.

f CR: individual circadian rhythm component.

## Discussion

### Principal Findings

This cross-sectional analysis was conducted to determine if a set of detailed accelerometry-derived summary measures of physical activity and circadian rhythmicity distinguishes between MS subtypes. As an extension of the work we and others had previously published, in which physical activity counts were on average lower in those with MS who were more disabled [9-11], we show here that participants with PMS exerted less total daily volume of physical activity than those with RRMS, and that these differences seemed more pronounced during specific times of the day. Sensitivity analyses to account for differences in timing and duration of sleep using the accelerometry metrics during the M10 period demonstrated very similar findings, which, if confirmed in subsequent studies, might help improve the feasibility of data collection (eg, if participants are not eager to wear the device overnight).

This work newly demonstrates that beyond average overall activity measures, specific PA indices had stronger discriminative properties than others in distinguishing PMS from RRMS. For example, those with PMS were more likely to transition from active to nonactive behavior (higher ASTP) and had less time spent in MVPA, instead favoring a greater amount of time in which they are inactive or only lightly active. ASTP may be a complementary measure of functionality to the measures of volume of daily activity such as TAC and MVPA. In the general population, higher ASTP is associated with worse health and functional status and higher fatiguability in older adults [20]. Our findings are consistent, as people with PMS have overall greater disability and symptoms. Therefore, it may be useful for future studies to explore novel measures of fragmentation of physical activity to determine indices that could better distinguish MS subtype.

Although differences in patterns of activity were demonstrated between the PMS and RRMS subgroups, exploring accelerometry-measured sleep and circadian/diurnal rhythmicity may discern even more significant differences. For example, we previously demonstrated that among people with MS, diurnal variability is greater in those with higher EDSS [9]. Herein, we confirmed that differences in TAC within specific times of the day (8 AM to 12 PM; 2 PM to 8 PM) appeared to drive the daily average activity measures. Participants with PMS also had higher IV and lower IS, further demonstrating the importance of detecting differences in diurnal rhythmicity between the 2 groups. The observed differences in our cohort herein serve as motivation for further exploration with more advanced analytical techniques beyond the use of averages of daily CR measures. Our next steps will include further investigation of more complex measures of fragmentation of physical activity, distributional and temporally local distributional characteristics of activity, and parameters of sleep and circadian disruptions [19,28,30-32].

An application of JIVE demonstrated a significant overlap between variation in physical activity and circadian rhythmicity domains. Joint component 1 was significant and was primarily driven by the total volume of PA along with measures of strength of CR (M10, fPC1, MESOR, amplitude), suggesting that a joint study of these 2 domains may provide a more accurate picture and capture the interdependence between the domains and their combined association with MS subtype. The significant association of MS subtype with the individual PA-1 and PA-2 components confirmed the results from the standalone associations of individual measures with MS subtype. Specifically, more fragmented physical activity profiles with lower times spent in MVPA and LIPA can be used to differentiate PMS and RRMS.

Accelerometry did not distinguish between the RRMS subgroups cross-sectionally. Correctly classifying people with MS as having transitioned from RRMS to PMS often takes several years and is usually done retrospectively [33]. In designing this cohort, we hypothesized that data acquired in the early phase of cohort follow-up would predict subsequent worsening as measured by EDSS and that accelerometry may help to reduce the time of diagnostic uncertainty. Since we report only cross-sectional data, we did not expect substantial differences between the RRMS groups, especially as these groups were matched for key characteristics (age, sex, race or ethnicity, and disease modifying therapy class). Longitudinal tracking of the participants will allow for tracking within-person changes in disability trajectories. Whether including additional features of accelerometry data, such as more advanced measures of sleep or circadian rhythm, will better distinguish RRMS subpopulations even at a single time point will be an important next step.

Finally, although our findings are currently not immediately applicable in clinical practice, the study suggests it may be possible to alert clinicians about certain patterns in 24-hour behaviors that may affect the lives of people with MS. Though it can be difficult to clinically distinguish PMS from RRMS, accelerometry measures appear to detect distinct patterns of activity. People with PMS seem to have increased variability and fragmentation of their activity patterns, and intermittently reduced function is commonly reported, subjectively, by people with more advanced MS during clinical visits. This increases the confidence that accelerometry can in fact measure clinically meaningful patterns in people with MS. It is important for future efforts to recapitulate these findings with commercial accelerometers in order to support scalability and to translate the findings into results that clinicians and patients can easily understand and monitor over time. For instance, the increased variability in people with PMS indicates that their experience of their disease may be very different from the clinical picture evident during their clinical visits. A discussion with patients regarding their physical activity, supplemented by accelerometry data, could shed light on other aspects of their lives that should be acknowledged. Understanding when patients are likely to be at their peak activity levels may help plan their day better (when to plan effortful tasks or schedule breaks) and may inform lifestyle modifications such as exercise or even physical therapy interventions to evaluate for improvements.

### Limitations

There are limitations to the study. The classification of RRMS as stable versus suspected progression is subjective, and it is likely that some of these individuals were misclassified, where a stable person was deemed as having suspected progression or vice versa, which could contribute to the lack of ability to detect differences in accelerometry measures between the 2 RRMS groups. We chose to create this subclassification to mirror the “clinical intuition” whereby physicians and, often, their patients worry they are progressing even if they do not meet the criteria for sustained EDSS worsening, and we wanted to be sure that sufficient numbers of such people were enrolled in the study. The longitudinal trajectories of the participants with RRMS will be informative; since all are at risk, based on age, of transitioning to a PMS phenotype, we can evaluate this risk as it relates to accelerometry measures not only using these subjective subtypes but also using a more agnostic, data-driven approach within the participants with RRMS subgroup as a whole. Another potential bias related to our study is the Hawthorne effect, where participants could potentially alter their physical activity because they were being monitored. Although this risk cannot be eliminated, it was minimized by disabling the features that display activity levels on the ActiGraph monitors (ie, the number of steps); only the time of day was displayed. It is possible that relevant comorbidities were missed during chart review although this is unlikely to affect a substantial enough proportion of individuals to meaningfully have influenced results. Finally, this is a cross-sectional study, and we will be able to better explore the dynamic changes in activity measured by accelerometry as well as fluctuations in the EDSS with the longitudinal follow-up, which will provide deeper insight into the evolution of MS progression measures.

### Conclusions

In conclusion, accelerometry-derived measures captured differences in physical activity and circadian rhythm profiles between people with PMS and RRMS. Further analytic approaches that consider more sophisticated approaches to time (such as circadian patterns) may help further refine the ability to detect differences between these subgroups at a single time point, whereas longitudinal follow-up is most likely to allow for the identification of accelerometry patterns that portend subsequent disability worsening as defined by EDSS. We specifically hypothesize that the measures that distinguish PMS from RRMS at baseline will be those to change first in the participants with RRMS who transition to PMS in the longitudinal follow-up of this cohort. Enabling the identification of MS progression or risk may help with clinical decision-making (such as choosing an appropriate disease-modifying therapy for a given person) or may enable advancements in telemedicine by tracking functions remotely. More importantly, these technologies will likely facilitate therapeutic development for PMS by improving disability detection and worsening, alone or in combination with other metrics such as neuroimaging, which will enable faster and smaller trials of promising interventions.

## Supplementary material

10.2196/57599 Multimedia Appendix 1 Illustrations of the study framework along with additional statistical analyses and sensitivity checks.

## Abbreviations

ASTP: active-to-sedentary transition probability
CR: circadian rhythm
DARE: daytime activity ratio estimate
EDSS: Expanded Disability Status Scale
fPC: functional principal component
HEAL-MS: home-based evaluation of actigraphy to predict longitudinal function in multiple sclerosis
IPAQ: International Physical Activity Questionnaire
IS: interdaily stability
IV: intradaily variability
JIVE: Joint and Individual Variation Explained
L5: average log acceleration during the least active 5 hours of the day
LIPA: light intensity physical activity
M10: average log acceleration during the most active 10 hours of the day
MESOR: midline estimating statistic of rhythm
MS: multiple sclerosis
MVPA: moderate to vigorous physical activity
NHANES: National Health and Nutrition Examination Survey
PA: physical activity
PMS: progressive multiple sclerosis
RA: relative amplitude
REDCap: Research Electronic Data Capture
RRMS: relapsing-remitting multiple sclerosis
SATP: sedentary-to-active transition probability
TAC: total activity count
TLAC: total log-transformed activity count
