# Supplementary material for: Accelerometry-Assessed Physical Activity and Circadian Rhythm to Detect Clinical Disability Status in Multiple Sclerosis: Cross-Sectional Study
Source: JMIR Mhealth Uhealth. 2025 Mar 31;13:e57599. doi: 10.2196/57599 (PMC11975255; doi:10.2196/57599)
Supplement: Multimedia Appendix 1 [file mhealth-v13-e57599-s001.docx]

**Table S1.** Minutes spent in MVPA, LIPA, and non-active (sleep and sedentary behaviors) for all participants.

|  | **Median [Interquartile range]** |
| --- | --- |
| MVPA minutes | 45 [0,120] |
| LIPA minutes | 300 [0, 510] |
| Non-active minutes | 1010 [800, 1420] |

MVPA: moderate-to-vigorous physical activity; LIPA: light intensity physical activity.

The following sample specific cut points were developed for discriminating between non-active, LIPA, and MVPA minutes: a) non-active minute was defined as a minute with activity counts ≤ 2000; b) LIPA minute was defined as activity counts > 2000 and ≤ 6750; c) MVPA minute was defined as activity counts > 6750. Cut points were specific to our cohort and defined based on an internal calibration procedure that maximized concordance between accelerometry-based estimates and the participants’ self-reported physical activity outcomes in the IPAQ. Note that this procedure is similar to recent developments for national level cohorts [22]. The threshold of >6750 activity counts for MVPA was determined from the self-reported minutes spent in moderate/vigorous activity on the IPAQ questionnaires. Only data from subjects who reported non-zero minutes for MVPA were used. After combining data for all subjects across all days, the value of 6750 activity counts corresponded to the daily median of 45 minutes for MVPA across all subjects in self-reported non-zero MVPA. For the threshold between LIPA and Non-activity, cut-points were defined using matching-procedure to maximize concordance with age and gender matched nationally representative references [22]. This approach led to the cut point of 2000 activity counts per minute to discriminate LIPA/non-active minutes.

**Table S2.** Mean (SD) baseline accelerometry metrics for HEAL-MS participants in the M10 period.

|  | **RRMS** | | **PMS**  **(N=85)** | ***P* value^a^** |
| --- | --- | --- | --- | --- |
|  | **RRMS-Stable (N=86)** | **RRMS-Suspected progression (N=82)** |  |  |
| TAC (x10^3^) | 1489 (399) | 1524 (506) | 1297 (484) | **<.001** |
| TLAC | 3898 (465) | 3908 (463) | 3779 (513) | .054 |
| Non-active minutes | 334 (70) | 327 (75) | 357 (87) | **.01** |
| MVPA | 46 (27) | 47 (40) | 31 (32) | **<.001** |
| LIPA | 220 (57) | 226 (51) | 212 (69) | .18 |
| TAC 1^b^ (x10^3^) | 344 (107) | 367 (123) | 303 (117) | **<.001** |
| TAC 2^b^ (x10^3^) | 286 (87) | 298 (108) | 256 (102) | **.007** |
| TAC 3^b^ (x10^3^) | 283 (95) | 276 (100) | 243 (96) | **.005** |
| TAC 4^b^ (x10^3^) | 281 (86) | 280 (107) | 239 (98) | **.001** |
| TAC 5^b^ (x10^3^) | 296 (88) | 302 (119) | 256 (99) | **.002** |
| SATP | 0.20 (0.07) | 0.20 (0.07) | 0.18 (0.07) | **.04** |
| ASTP | 0.24 (0.06) | 0.23 (0.06) | 0.27 (0.11) | **<.001** |

TAC: total activity count; TLAC: total log-transformed activity count; MVPA: moderate to vigorous physical activity; LIPA: light intensity physical activity; DARE: daytime activity ratio estimate; SATP: sedentary to active transition probability; ASTP: active to sedentary transition probability.

^a^ p-value from t-test comparing RRMS (RRMS-Stable and RRMS-Suspected progression) vs PMS.

^b^ TAC 1-5 represent TAC divided into five 2-hour intervals within M10 (i.e., TAC 1 represents the first two hours in the 10-hour M10 interval for each participant; TAC 2 represents the second two hours in the M10 interval, etc).

**Table S3.** Multivariable logistic regression model to evaluate the association of each accelerometry metric within the M10 period with MS subtype.

|  | **PMS vs RRMS (reference group)** | | | **RRMS-Suspected progression vs RRMS-Stable (reference group)** | | |
| --- | --- | --- | --- | --- | --- | --- |
|  | Estimate | *P* value | 95% CI | Estimate | *P* value | 95% CI |
| TAC^a^ | -0.47 | **.003** | -0.78, -0.17 | 0.08 | .63 | -0.26, 0.43 |
| TLAC^a^ | -0.23 | .10 | -0.50, 0.05 | 0.03 | .86 | -0.30, 0.37 |
| Non-active minutes | 0.004 | **.02** | 0.0007, 0.008 | -0.001 | .53 | -0.006, 0.003 |
| MVPA | -0.02 | **.002** | -0.03, -0.007 | 0.0009 | .86 | -0.009, 0.01 |
| LIPA | -0.003 | .21 | -0.008, 0.002 | 0.002 | .48 | -0.004, 0.008 |
| TAC 1^a,b^ | -0.47 | **.002** | -0.79, -0.18 | 0.23 | .18 | -0.10, 0.58 |
| TAC 2^a,b^ | -0.38 | **.01** | -0.69, -0.10 | 0.12 | .47 | -0.20, 0.45 |
| TAC 3^a,b^ | -0.38 | **.01** | -0.68, -0.08 | -0.08 | .63 | -0.41, 0.25 |
| TAC 4^a,b^ | -0.43 | **.006** | -0.74, -0.13 | 0.001 | .99 | -0.33, 0.34 |
| TAC 5^a,b^ | -0.44 | **.005** | -0.76, -0.14 | 0.06 | .74 | -0.27, 0.39 |
| SATP | -3.19 | .14 | -7.54, 0.98 | 0.39 | .87 | -4.24, 5.05 |
| ASTP | 6.54 | **.0008** | 2.91, 10.58 | -3.03 | .27 | -8.64, 2.22 |
| MVPA/LIPA | -17.88 | **.002** | -29.69, -7.25 | 0.50 | .92 | -9.97, 11.23 |
| MVPA/Non-active | -1.90 | .052 | -3.96, -0.19 | 0.76 | .34 | -0.57, 2.56 |
| LIPA/Non-active | -0.15 | .20 | -0.39, 0.07 | 0.14 | .27 | -0.11, 0.41 |

TAC: total activity count; TLAC: total log-transformed activity count; MVPA: moderate-to-vigorous physical activity; LIPA: light intensity physical activity; DARE: daytime activity ratio estimate; SATP: sedentary to active transition probability; ASTP: active to sedentary transition probability.

The multivariate logistic regression models were adjusted for age, sex, and body mass index.

^a^ Regression inputs were scaled by dividing TAC and TLAC values by one standard deviation.

^b^ TAC 1-5 represent TAC divided into five 2-hour intervals within M10 (i.e., TAC 1 represents the first two hours in the 10-hour M10 interval for each participant; TAC 2 represents the second two hours in the M10 interval, etc).

**Table S4.** Individual JIVE components of circadian rhythm with variables with more than 5% of proportional variation.

| **Variable** | **CR Component Loading** | **Relative Magnitude of CR Component Loading** |
| --- | --- | --- |
| **Circadian Rhythm Component 1** | | |
| DARE | -0.4363715 | 0.1904201 |
| Acrophase | 0.3898568 | 0.1519883 |
| Midpoint of M10 | 0.3295719 | 0.1086176 |
| fPC 2 | 0.3161498 | 0.0999507 |
| fPC 3 | 0.3049699 | 0.0930066 |
| Midpoint of L5 | 0.2964383 | 0.0878756 |
| IS | -0.2666920 | 0.0711246 |
| RA | -0.2564089 | 0.0657455 |
| L5 | 0.2302475 | 0.0530139 |
| **Circadian Rhythm Component 2** | | |
| RA | 0.4249533 | 0.1805853 |
| L5 | -0.4196557 | 0.1761109 |
| fPC 2 | 0.3742011 | 0.1400265 |
| Midpoint of M10 | 0.3486876 | 0.1215830 |
| Midpoint of L5 | 0.3199200 | 0.1023488 |
| Acrophase | 0.3167421 | 0.1003256 |
| **Circadian Rhythm Component 3** | | |
| fPC 3 | 0.5909505 | 0.3492225 |
| fPC 4 | -0.5477032 | 0.2999788 |
| fPC 5 | -0.3070923 | 0.0943057 |
| Midpoint of L5 | -0.3027682 | 0.0916686 |
| fPC 2 | -0.2337843 | 0.0546551 |
| Midpoint of M10 | 0.2239214 | 0.0501408 |

DARE: daytime activity ratio estimate; M10: average log acceleration during the most active 10 hours of the day; fPC: functional principal component; L5: log acceleration during the least active 5 hours of the day; IS: inter-daily stability; RA: relative amplitude.

**Figure S1.** Timeline of outcome measures collected during the 4.5 years of follow-up for the HEAL-MS study.


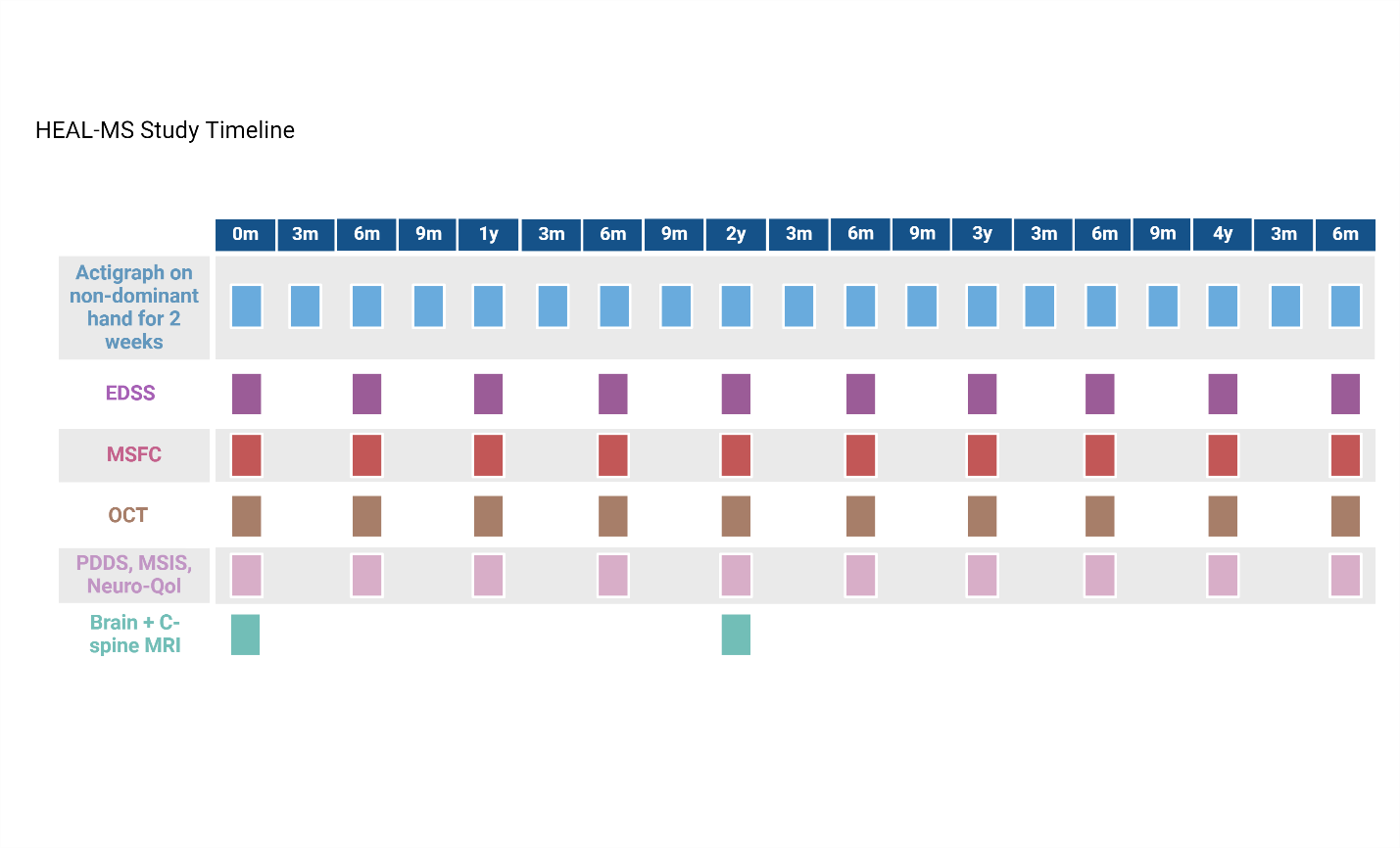


m: months; y: year; EDSS: Expanded Disability Status Scale; MSFC: Multiple Sclerosis Functional Composite; OCT: optical coherence tomography; PDDS: Patient-Determined Disease Steps; MSIS: MS Impact Score; Neuro-QoL: Quality of Life in Neurological Disorders; C-spine: Cervical spine.

0m = Baseline visit.

**Figure S2.** The distribution of Non-active, LIPA, and MVPA minutes across all participants when Non-active ≤ 2000 activity counts, 2000 > LIPA ≤ 6750, and MVPA > 6750.


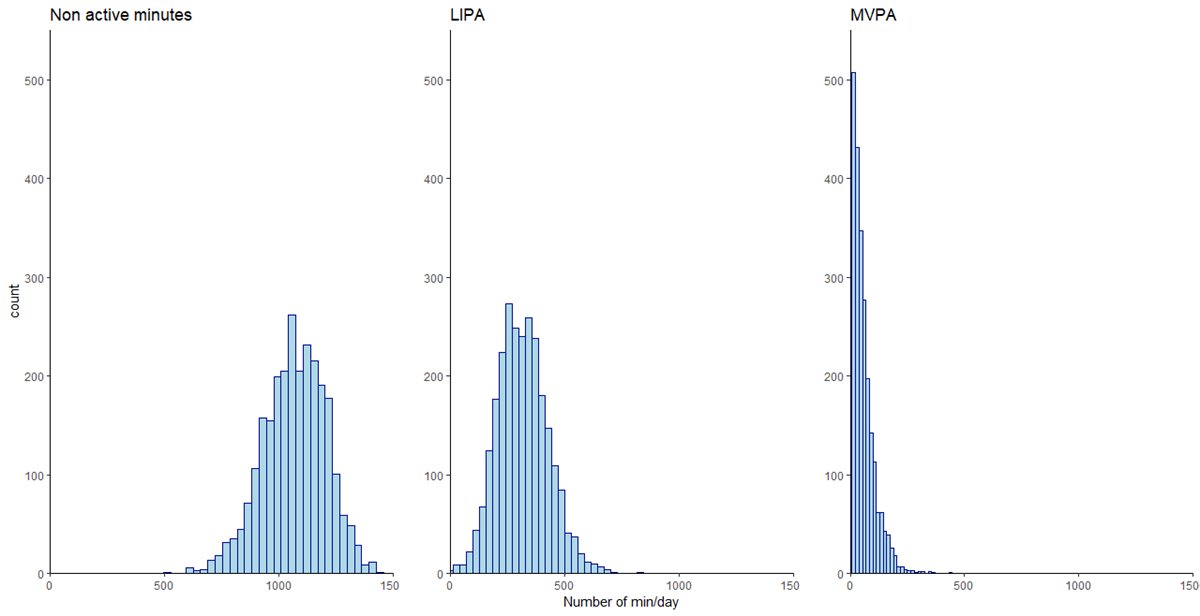


MVPA: moderate-to-vigorous physical activity; LIPA: light intensity physical activity.

**Figure S3.** Scree plot showing the variance explained by the functional principal components (fPCs).


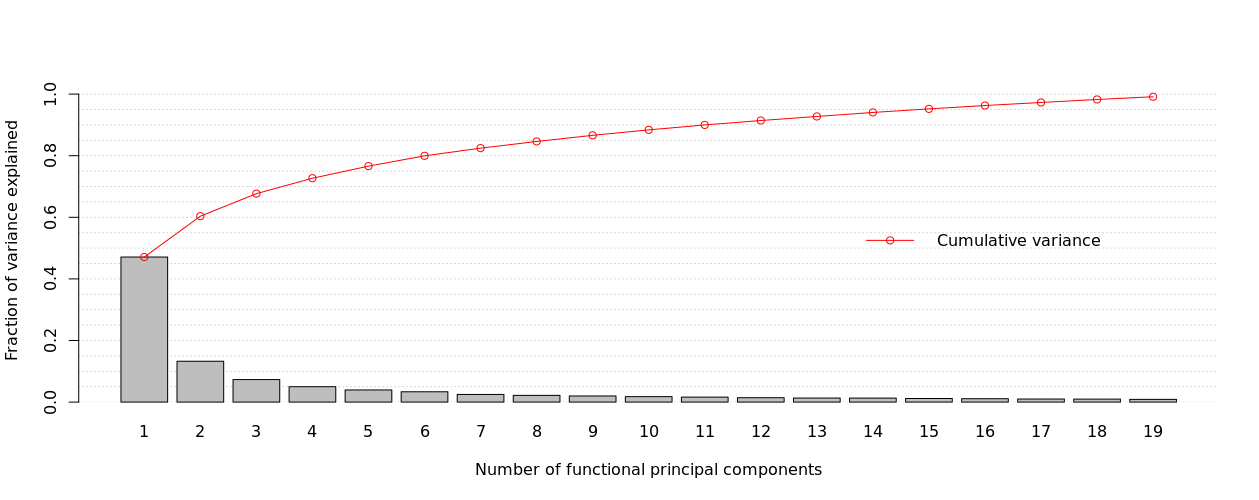


**Figure S4.** Raw accelerometry data for one participant over the course of seven days.


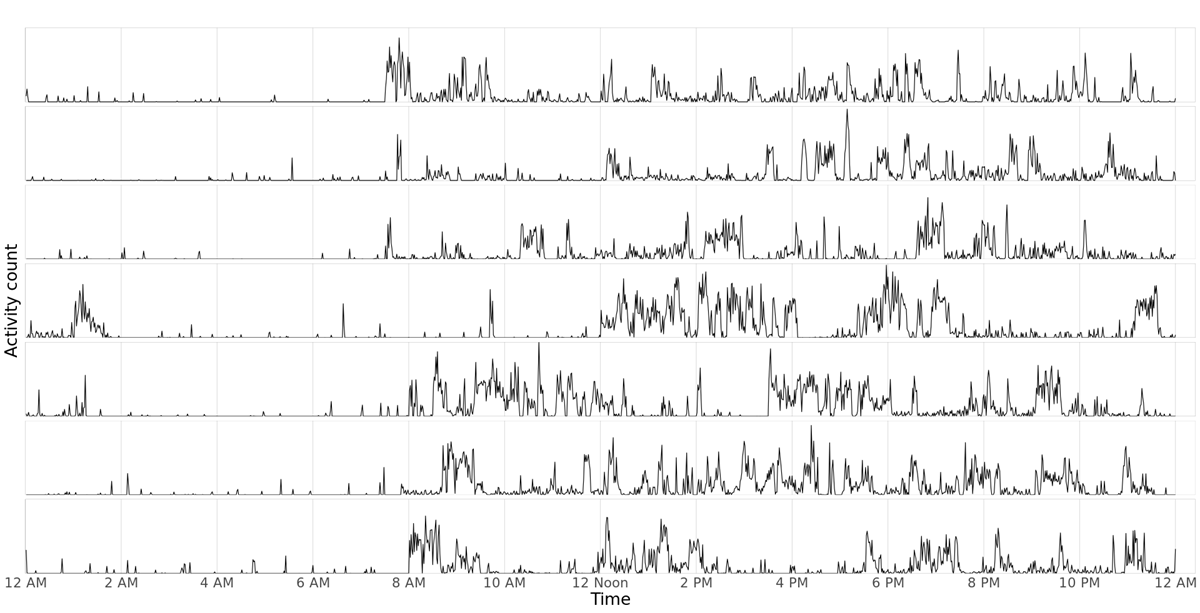


**Figure S5.** Correlation plot of the accelerometry-derived metrics.


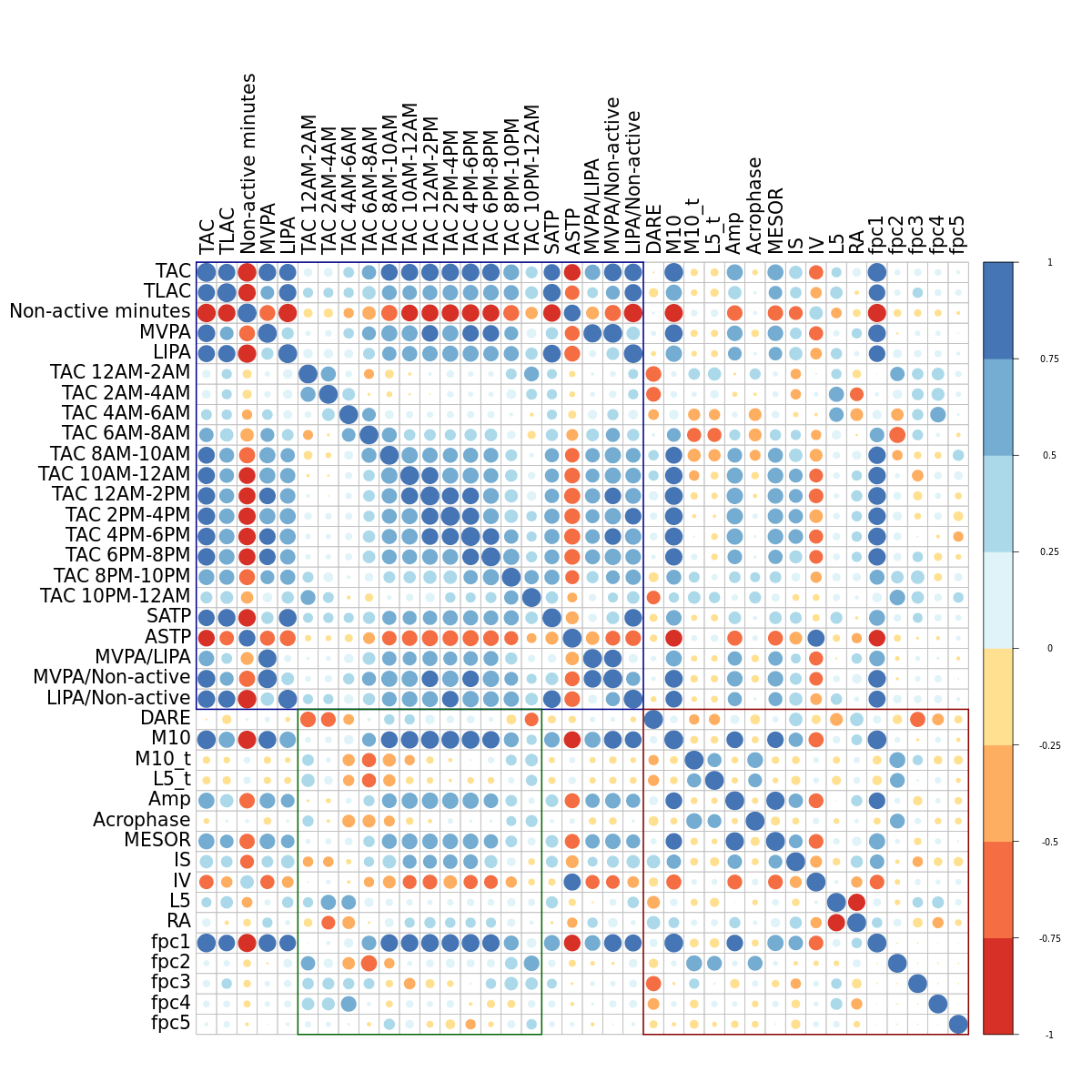


TAC: total activity count; TLAC: total log-transformed activity count; MVPA: moderate-to-vigorous physical activity; LIPA: light intensity physical activity; SATP: sedentary to active transition probability; ASTP: active to sedentary transition probability; DARE: daytime activity ratio estimate; M10: average log acceleration during the most active 10 hours of the day; L5: log acceleration during the least active 5 hours of the day; Amp: amplitude; MESOR: mid-line estimating statistic of rhythm; IS: inter-daily stability; IV: intra-daily variability; RA: relative amplitude; fPC: functional principal component.

The blue box shows the correlation of the physical activity measures. The red box shows the correlation of the circadian rhythm measures. The green box shows the correlation of circadian rhythm accelerometry measures with total activity counts (TAC) over 24 hours.

**Figure S6.** Total activity counts (TAC) for each 2-hour interval over the course of 24 hours for RRMS-Stable vs RRMS-Suspected progression.


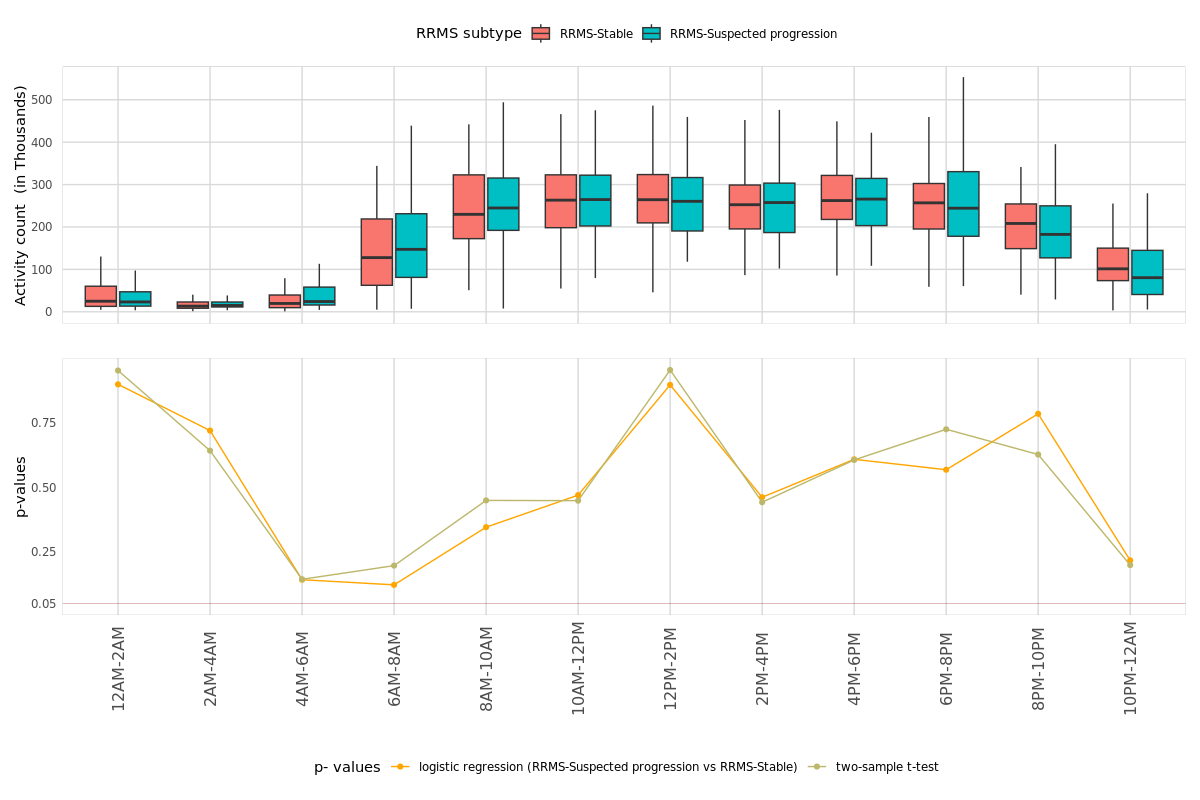


The two-sample t-test p-values show the 2-hour intervals when TAC was significantly different between the two groups.

The logistic regression p-values show the 2-hour intervals when TAC was significantly associated with odds of RRMS-Suspected progression (vs RRMS-Stable), after adjusting for age, sex, and body mass index.
